# Sustained overexpression of spliced X-box-binding protein-1 in neurons leads to spontaneous seizures and sudden death in mice

**DOI:** 10.1038/s42003-023-04594-8

**Published:** 2023-03-09

**Authors:** Zhuoran Wang, Qiang Li, Brad J. Kolls, Brian Mace, Shu Yu, Xuan Li, Wei Liu, Eduardo Chaparro, Yuntian Shen, Lihong Dang, Ángela del Águila, Joshua D. Bernstock, Kory R. Johnson, Junjie Yao, William C. Wetsel, Scott D. Moore, Dennis A. Turner, Wei Yang

**Affiliations:** 1 Multidisciplinary Brain Protection Program, Department of Anesthesiology, Duke University Medical Center, Durham, NC USA; 2 Department of Neurology, Duke University Medical Center, Durham, NC USA; 3 Department of Bioengineering, Duke University, Durham, NC USA; 4 Department of Neurosurgery, Duke University Medical Center, Durham, NC USA; 5 National Institute of Neurological Disorders and Stroke, NINDS/NIH, Bethesda, MD USA; 6 Departments of Neurobiology and Cell Biology, Duke University Medical Center, Durham, NC USA; 7 Department of Psychiatry and Behavioral Sciences, Duke University Medical Center, Durham, NC USA; 8 Departments of Neurosurgery, Neurobiology and Biomedical Engineering, Duke University Medical Center, Durham, NC USA

**Keywords:** Epilepsy, Epilepsy

## Abstract

The underlying etiologies of seizures are highly heterogeneous and remain incompletely understood. While studying the unfolded protein response (UPR) pathways in the brain, we unexpectedly discovered that transgenic mice (XBP1s-TG) expressing spliced X-box–binding protein-1 (*Xbp1s*), a key effector of UPR signaling, in forebrain excitatory neurons, rapidly develop neurologic deficits, most notably recurrent spontaneous seizures. This seizure phenotype begins around 8 days after *Xbp1s* transgene expression is induced in XBP1s-TG mice, and by approximately 14 days post induction, the seizures evolve into status epilepticus with nearly continuous seizure activity followed by sudden death. Animal death is likely due to severe seizures because the anticonvulsant valproic acid could significantly prolong the lives of XBP1s-TG mice. Mechanistically, our gene profiling analysis indicates that compared to control mice, XBP1s-TG mice exhibit 591 differentially regulated genes (mostly upregulated) in the brain, including several GABA_A_ receptor genes that are notably downregulated. Finally, whole-cell patch clamp analysis reveals a significant reduction in both spontaneous and tonic GABAergic inhibitory responses in *Xbp1s*-expressing neurons. Taken together, our findings unravel a link between XBP1s signaling and seizure occurrence.

## Abstract (web summary)

XBP1s is a key effector of the unfolded protein response. Neuron-specific overexpression of *Xbp1s* in the brain leads to impaired GABAergic signaling and recurrent spontaneous seizures in mice.

## Introduction

Spontaneous seizures are frequently observed in neurologic disorders. The underlying etiologies of seizures are highly heterogeneous (e.g., brain injuries and genetic origins), and remain poorly understood^1^. While analyzing the roles of the unfolded protein response (UPR) in brain function, we unexpectedly discovered that transgenic mice, which conditionally express spliced X-box–binding protein-1 (*Xbp1s*) in forebrain excitatory neurons, displayed severe recurrent spontaneous seizures, leading to sudden death over a short but predictable time period.

In general, the UPR is activated when unfolded/misfolded proteins accumulate in the endoplasmic reticulum (ER), and ER function is compromised, a state termed ER stress^2^. Activation of the UPR to restore ER homeostasis is critical to cell survival and organismal health because the ER is the subcellular compartment where newly synthesized membrane and secretory proteins are folded and processed. Among the 3 UPR branches, the most evolutionarily conserved branch is controlled by the stress sensor protein inositol-requiring enzyme-1 (IRE1). Upon activation, IRE1 functions as an endonuclease that unconventionally cleaves *Xbp1* mRNA to generate spliced *Xbp1* mRNA, which is then translated into XBP1s protein, a transcription factor. XBP1s can upregulate expression of genes coding for ER-resident chaperones and proteins involved in ER-associated degradation of misfolded proteins, thereby helping to reduce ER stress and re-establish cellular protein homeostasis (proteostasis)^3^. Indeed, studies have shown that overexpression of *Xbp1s* could be beneficial. For example, *Xbp1s* overexpression suppresses β-amyloid neurotoxicity in an Alzheimer’s disease fly model^4^. Cisse and colleagues used a viral-mediated gene-delivery approach to overexpress *Xbp1s* in the hippocampus of Alzheimer’s disease mice, and found that it markedly improved cognitive function in these mice^5^. Further, overexpression of *Xbp1s* in neurons or glial cells of *C. elegans* has been reported to improve proteostasis in multiple organs, resulting in an increase in longevity^6,7^. However, XBP1s signaling induced by sustained ER stress can contribute to disease pathogenesis.

Critically, current evidence indicates that XBP1s is not only a key regulator of adaptive responses to ER stress, but can also exert non-canonical and ER stress-independent functions^8^. For example, the IRE1/XBP1 pathway is involved in neural development and regulation of neurotransmitter systems^9,10^. Moreover, *Xbp1s* overexpression in the brain appears to improve long-term memory, partly due to XBP1s-regulated alterations within the BDNF pathway^11^. Notably, the XBP1 pathway has also been implicated in psychiatric disorders. For example, a polymorphism in the promoter region of *XBP1* has been proposed to be a genetic risk factor for bipolar disorder in Japanese populations^12^. Together, these data strongly suggest that XBP1s is a unique multifunctional protein that controls a plethora of cellular functions, and its dysregulation may lead to aberrant states.

In our efforts to develop conditional and inducible transgenic mice overexpressing *Xbp1s* limited to forebrain excitatory neurons, we discovered that within a few days after inducing expression of *Xbp1s*, these adult transgenic mice began to exhibit progressively worsening seizures followed by death. A few human and animal observational studies have implicated a link between epileptic seizures and ER stress^13,14^. For example, in human epileptic brain samples and animal brains with induced seizures, UPR markers, including XBP1s, are upregulated, suggesting seizure-mediated perturbation of ER proteostasis^14^. The current view is that seizures lead to ER stress, and activate the UPR, which may then contribute to apoptotic neuronal death, mainly via the PERK-ATF4-CHOP axis^14^. Indeed, some compounds that can reduce ER stress have shown neuroprotective effects in animal seizure models^14^. However, what we observed in our *Xbp1s* transgenic mice is distinct from previous studies, as *Xbp1s* is overexpressed in neurons in the absence of ER stress. To date, no evidence has shown that forced activation of a UPR branch in the brain can cause seizures. Therefore, this study is designed to understand this unexpected phenotype.

We first found that post-treatment with an anticonvulsant drug partially rescued this severe phenotype. Our gene expression profiling analysis revealed that *Xbp1s* expression in neurons modulates a network involving the post-synaptic GABA_A_ receptor pathway. Consistent with this finding, our whole cell patch clamp recordings demonstrated a significant reduction in GABAergic inhibitory post-synaptic function in *Xbp1s*-expressing neurons. Together, our data unveiled a previously unrecognized role for XBP1s in the pathogenesis of spontaneous seizures through post-synaptic inhibitory alterations. These findings may have important clinical implications. For example, in a case report, 3 unrelated individuals—who were documented to have various types of seizures that were resistant to multiple drugs, and who failed to achieve developmental milestones—carried *RNF13* gain-of-function mutations, which leads to an increase in *XBP1s* expression^15^.

## Results

### Sustained overexpression of *Xbp1s* in forebrain neurons led to spontaneous seizures and lethality in mice

To study the role of XBP1s in brain function, we initially set out to generate conditional and inducible *Xbp1s* transgenic mice (XBP1s-TG) in which neuron-specific expression of *Xbp1s* is controlled by a Tet-off system with a Camk2a promoter (Supplementary Fig. 1a, b). At first, we bred mice with regular drinking water so that *Xbp1s* transgene expression was not suppressed. Unexpectedly, under this breeding condition, XBP1s-TG neonatal mice began to die shortly after birth, and no adult XBP1s-TG mice were obtained.

**Fig. 1:**
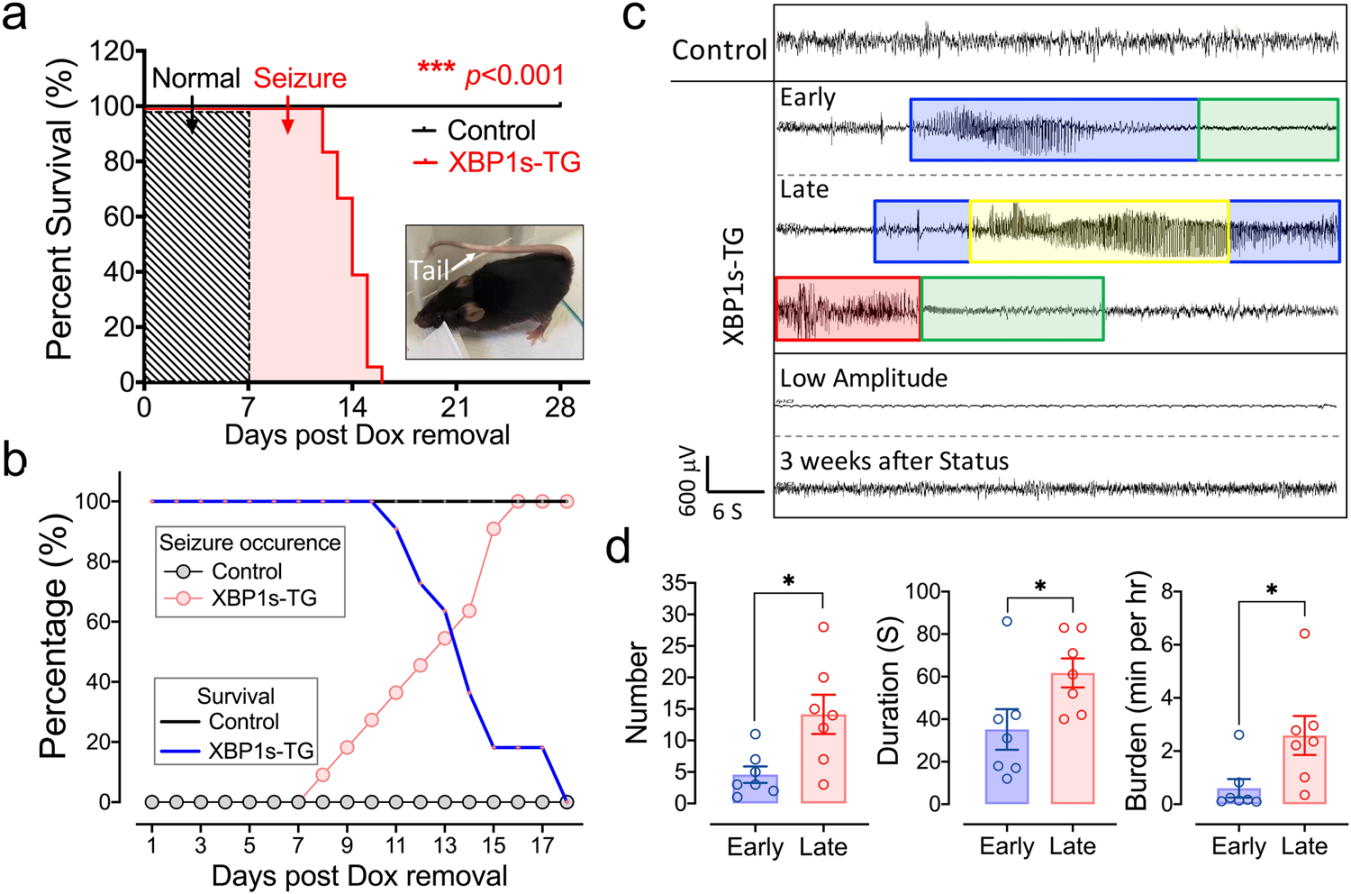
Sustained *Xbp1s* expression in neurons results in spontaneous seizures and death. **a** Survival of adult XBP1s-TG mice after induction of *Xbp1s* expression. XBP1s-TG and control mice were maintained with Dox in the drinking water prior to induction. When the mice were 2–3 months old, Dox was removed. The survival rate was recorded over 4 weeks. *n* = 17–18/group. The highlighted area labeled “seizure” refers to the presence of seizure behaviors. Inset: a representative picture of XBP1s-TG mice showing a seizure-like phenotype. **b**–**d** Progression of seizures with EEG monitoring. XBP1s-TG and control littermates were subjected to continuous EEG monitoring, immediately after removing Dox from the drinking water. **b** Seizure occurrence and survival rate. **c** Examples of EEG-recorded activity and seizures. The behavioral seizure activity is color-coded with blue boxes representing freezing, yellow boxes denoting rearing with automatisms of the forelimbs, and red boxes representing bounding behaviors. The period in the green box is a post-ictal period showing little to no movement. All the XBP1s-TG EEG recordings were from the same mouse. Of note, the two EEG traces at the bottom depict that the profoundly abnormal low amplitude EEG that was seen in several mice just hours before death was completely reversed to an essentially normal EEG pattern after adding Dox back to drinking water. **d** EEG characteristics of seizures. In the first 6 hours after seizure onset (early), the number, duration, and burden (time spent in seizures per hour) of seizures were lower during than during the late stage (i.e., the 6 hours just prior to transitioning to continuous seizure activity). Data are presented as mean ± SEM (*n* = 7/group). *p < 0.05; ***p < 0.001.

To determine whether this mortality was due to *Xbp1s* expression, we provided doxycycline (Dox) in the drinking water during the mating period to adulthood. Under this condition, all mice reached adulthood (up to 12 months of age in our observational period) without any apparent abnormal phenotype. However, when Dox was removed from the drinking water to induce *Xbp1s* transgene expression, some XBP1s-TG mice started to display seizure-like behavior under mild stimulation (e.g., mouse handling) as early as 7 days of Dox withdrawal. The presentation of spontaneous seizures (e.g., loss of upright posture and clonus of limbs) was progressive and by approximately day 9, all mice showed evident and spontaneous seizures, and then rapidly developed into more severe and frequent unprovoked behavioral seizures (Supplemental Movies 1, 2). Eventually, all XBP1s-TG mice died by approximately day 15 after Dox removal (Fig. 1a).

To better analyze the evolution of seizures in XBP1s-TG mice, we performed continuous video-electroencephalography (cvEEG) monitoring beginning 7 days after surgical placement of epidural EEG recording electrodes and continuing over 20 days. After Dox removal, the first seizures were observed as early as 8 days later in XBP1s-TG mice (Fig. 1b). The early seizures were characterized by behavioral freezing or arousal from sleep with little to no significant motor movement (Racine scores <3). Between days 8 and 13 post-Dox removal, all transgenic mice developed seizures while none of the controls had seizures. Within 3–4 days from the first seizure, the seizures became more frequent and longer in duration and eventually evolved into status epilepticus with nearly continuous seizure activity over a few hours before death. The behavioral correlates also became more severe over time, with the development of rearing, loss of balance, tonic-clonic movements, bounding behaviors, and eventually lying motionless on their sides in the cage (Racine scores ≥3).

Electrographically, early seizures occurred in 10–20 second periods of 4–5 Hz rhythmic activity that was symmetric and evolved into slower, post-ictal 2–3 Hz rhythmic activity post-ictally. Later seizures were longer, typically around 60 seconds in duration, had similar high-frequency onset, and evolved into high-amplitude 2 Hz sharp waves followed by longer periods of 1–2 Hz rhythmic delta. Some mice developed continuous electrographic seizure activity for several hours before death (status epilepticus). Other mice developed a profoundly abnormal low-amplitude EEG pattern for several hours just before death. Examples of the typical EEG patterns are shown in Fig. 1c. We quantified these electrographic changes by comparing the 6-hour period following the first seizure to the last 6-hour period just prior to the onset of continuous seizure activity or the low amplitude EEG (Fig. 1d). This analysis revealed that both seizure frequency and duration increased over time.

### Valproic acid treatment significantly prolonged the lifespan in XBP1s-TG mice

**Fig. 2:**
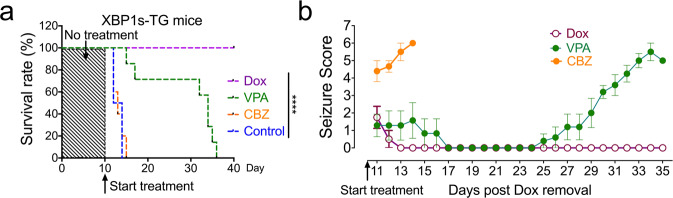
Pharmacologic interventions. On day 10 post-removal of Dox from the drinking water, XBP1s-TG mice (*n* = 4–7/group) received Dox in drinking water, valproic acid (VPA), or carbamazepine (CBZ) treatment. XBP1s-TG mice without any treatment served as control. **a** Survival rates (****p < 0.0001; Log-rank [Mantel-Cox] test). **b** Seizure scores (mean ± SEM).

To determine whether animal death was due to severe seizures, we assessed the therapeutic effects of 2 current anticonvulsant drugs, carbamazepine (CBZ) and valproic acid (VPA). XBP1s-TG mice were placed on drinking water without Dox for 10 days, which resulted in ongoing recurrent seizures. The animals were then randomly divided into 3 different treatment groups. In the first group, we put Dox back into the drinking water. Interestingly, although there were already seizure occurrences in XBP1s-TG mice by day 10 post induction, re-introduction of Dox to suppress transgene *Xbp1s* expression completely reversed the seizure and lethal phenotype. In the second group, CBZ treatment failed to demonstrate any major therapeutic effect in XBP1s-TG mice. Remarkably, in the third group, daily VPA treatment significantly extended the lifespan of XBP1s-TG mice (Fig. 2a). Seizure score data indicated that after adding Dox back in the drinking water, XBP1s-TG mice rapidly returned to seizure-free condition. For VPA treatment, seizure occurrence was suppressed for several days, and then gradually came back, eventually leading to animal death (Fig. 2b). Collectively, the mortality observed in XBP1s-TG mice was related directly to the severe seizures.

### Mapping the integration site of the *Xbp1s* transgene in XBP1s-TG mice

**Fig. 3:**
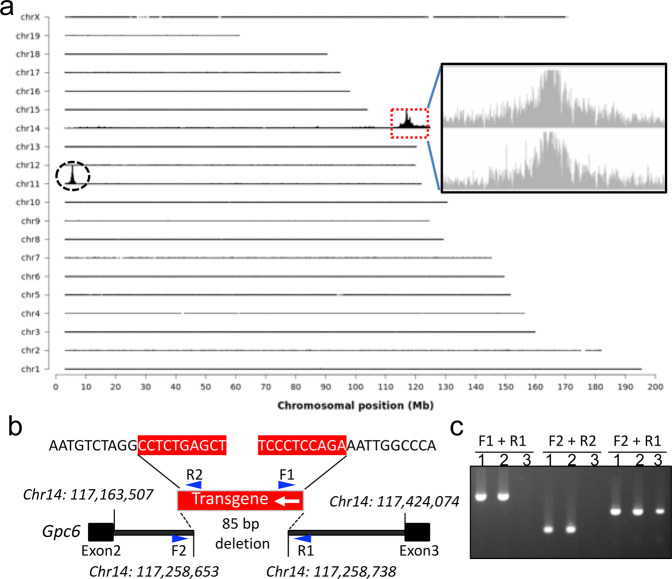
Integration site identification of the transgene in TRE-XBP1s mouse line. **a** Genome-wide TLA coverage. A detailed view of TLA sequence coverage surrounding the integration site (indicated by a red rectangle at chromosome 14) is shown in the inset. Peak at chromosome 11 shows endogenous *Xbp1* (indicated by a black circle). **b** Schematic of the insertion site. The transgene (red) was into the intron region between *Gpc6* exon 2 and exon 3. The blue arrows denote the primes for confirmation of the left and right junctions of the transgene. **c** PCR confirmation of the integration site. 1 and 2: TRE-XBP1s mouse DNA, and 3: wild-type mouse DNA. TLA targeted locus amplification.

Since the phenotype was totally unanticipated, we examined the possibility of whether the transgene insertion in TRE-XBP1s mouse line interferes with an endogenous gene, which then potentially contributes to the phenotype. To identify the exact transgene integration site(s), we used the targeted locus amplification (TLA) approach (Fig. 3). Our data indicated that there is only one insertion site in the TRE-XBP1s mouse line used in our study (Fig. 3a). Specifically, TLA analysis mapped the transgene insertion site to mouse chromosome 14, located in an intron of *glypican 6* (*Gpc6*) gene, with an 85 bp deletion at the integration site (Fig. 3b). This insertion site was confirmed by PCR analysis (Fig. 3c). Since the *Xbp1s* transgene is inserted in an intron, RNA-seq analysis indicated unaltered expression of *Gpc6* (see below), and no evidence indicates that *Gpc6* is a seizure-related gene, these data strongly support that the seizure phenotype observed in XBP1s-TG mice was caused by *Xbp1s* expression.

### Characterization of XBP1s-TG mice revealed extensive changes in neurologic functions, neuroinflammation, and physiology

**Fig. 4:**
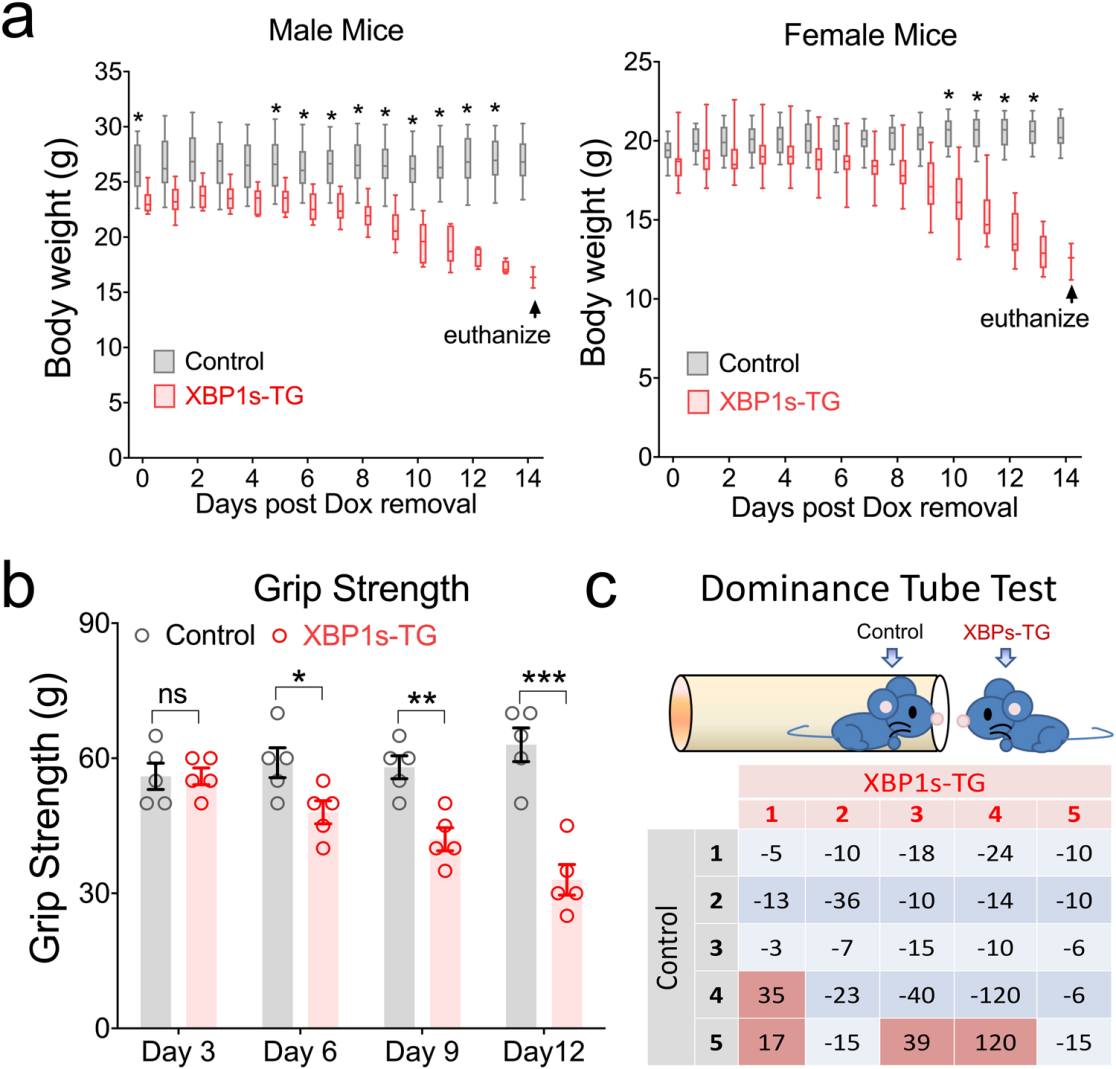
Behavioral phenotype in XBP1s-TG mice. **a** Body weight loss. Body weight of adult male (*n* = 6/group; RMANOVA: genotype effect [F(1,9) = 12.67, *p* = 0.006]) and female (*n* = 11–13/group; RMANOVA: genotype effect [F(1,12) = 12.56, *p* = 0.004]) mice were monitored after Dox removal. **b** Grip strength test. Mice were tested on the indicated days post-removal of Dox. Data are presented as mean ± SEM (*n* = 5/group). **c** Social dominance tube test (*n* = 5/group). Control and XBP1s-TG mice were kept on drinking water without Dox for 8 days. The latency time (in seconds) of XBP1s-TG mice are shown in the table. If XBP1s-TG mouse won, the latency time is positive (red). If XBP1s-TG mouse lost, the latency time is negative (blue-grey). *p < 0.05; **p < 0.01; ***p < 0.001.

To better understand this mouse line, we first examined general changes in overall health status, including physical and mental health, in XBP1s-TG mice. We found that a substantial loss in body weight was observed in XBP1s-TG mice after Dox removal (Fig. 4a). Results from the grip strength test indicated a progressive functional decline in muscle strength in XBP1s-TG mice, after the onset of *Xbp1s* expression (Fig. 4b). In the social dominance tube test, XBP1s-TG mice failed 84% of matches (Fig. 4c), suggesting more submissive compared to controls.

On day 8 after *Xbp1s* induction, no apparent abnormalities were identified in Nissl-stained brain sections of XBP1s-TG mice (Supplementary Fig. 1c). However, on day 13 after Dox removal, immunostaining analysis revealed dramatic activation of astrocytes (GFAP) and microglia (IBA1), denoting massive neuroinflammation in the XBP1s-TG brains (Supplementary Fig. 2). Together, these data indicate that sustained expression of *Xbp1s* in forebrain neurons severely deteriorated brain function, which is likely a consequence of the XBP1s-mediated seizures.

Metabolic changes can result in seizures, particularly low glucose levels. Current literature has documented that UPR signaling, especially the IRE1/XBP1s branch, plays a critical role in regulating metabolic processes such as glucose metabolism^3^. In line with previous findings, we observed a significant increase in blood glucose levels of Xbp1-cKO mice (Supplementary Fig. 3a). Consistently, we observed a decrease in blood glucose levels at day 6 after induction of *Xbp1s* expression (Supplementary Fig. 3b); however, no significant difference in glucose levels in the brain was found between wild-type and XBP1s-TG mice (Supplementary Fig. 3c). Further, we used functional photoacoustic microscopy (PAM) to image brains and assess the oxygen supply in the brain at a late stage in seizure progression. The PAM data showed that O_2_ saturation in brain vessels was comparable between control and XBP1s-TG mice on day 12 following the removal of Dox (Supplementary Fig. 4). Finally, there was no significant difference in blood gas levels between the genotypes (Supplementary Table 1). Thus, our findings did not support that *Xbp1s* overexpression disrupts energy metabolism (e.g., severe hypoglycemia), and thereby leads to seizures in XBP1s-TG mice.

### XBP1s is not only a master regulator of the UPR, but is also critically involved in the GABA_A_ pathway

**Fig. 5:**
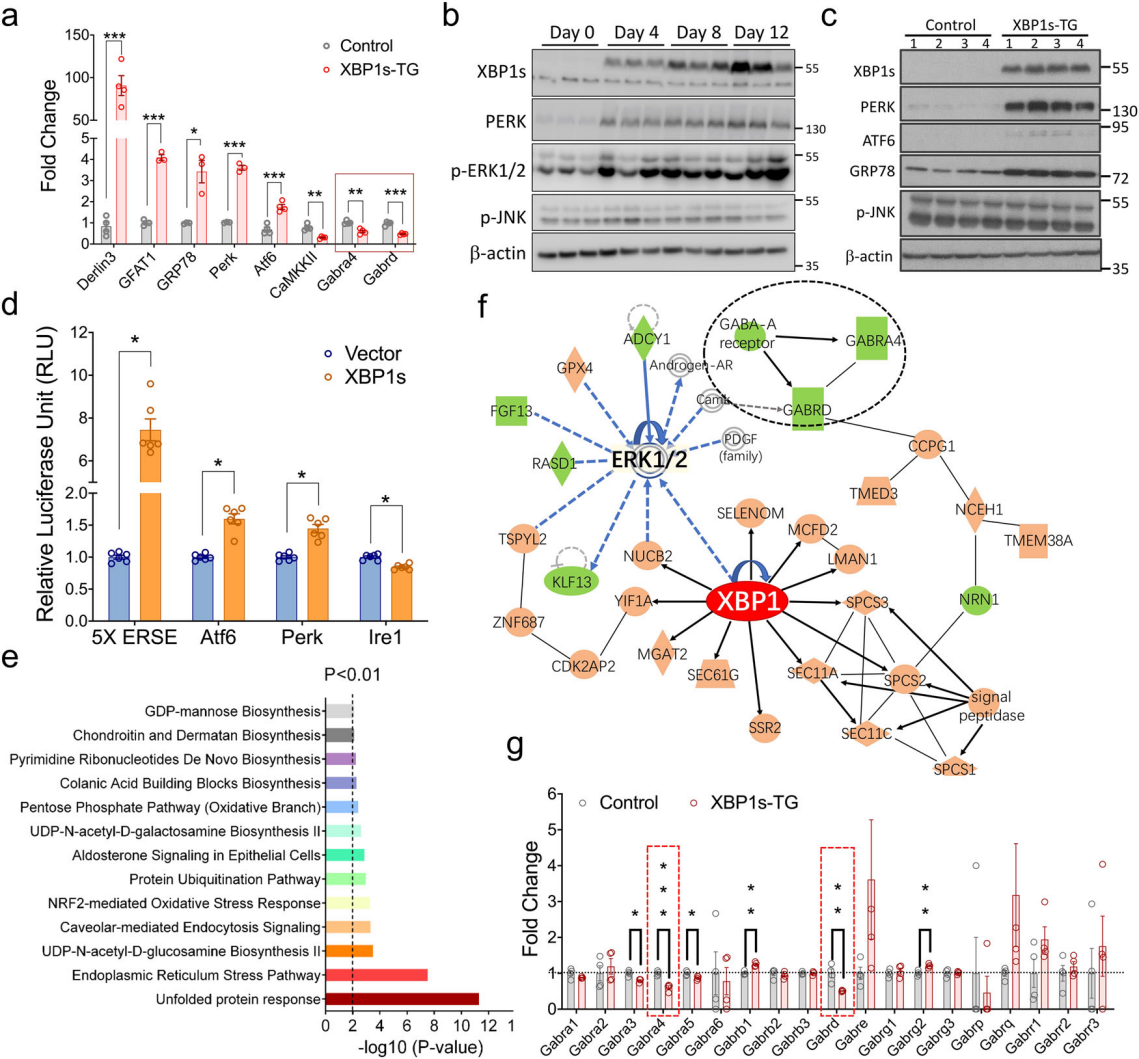
RNA-Seq analysis of hippocampal samples from control and XBP1s-TG mice. **a** Verification of RNA-Seq data by quantitative RT-PCR analysis. All data were normalized to β-actin. To calculate fold change, the mean values of control samples were set to 1.0. Data are presented as means ± SEM (*n* = 3/group). **b**–**d** Effects of XBP1s on UPR signaling. Western blotting analysis of key components of the UPR was performed using hippocampal samples. Brain samples were collected on the indicated days (**b**) or day 8 (**c**) after Dox removal from the drinking water. **d** Luciferase reporter analysis. Various promoter reporter vectors with empty vector or the *Xbp1s* expression vector were co-transfected into HEK293T cells. Luciferase activity was measured and compared. **e** IPA pathway analysis. **f** The predicted network. XBP1 is shown in red indicating high overexpression; brown indicates upregulated genes, while green signifies down-regulated genes. **g** RNA-Seq data of all subunits of the GABA receptors. The expression levels for the *Gabra4* and *Gabrd* subunits are marked by red dotted rectangles. Data are presented as mean ± SEM. *p < 0.01; **p < 0.01; ***p < 0.001.

Because XBP1s is a potent transcription factor, we then analyzed XBP1s-induced molecular consequences by gene profiling (Fig. 5). RNA-Seq analysis was performed on the hippocampal samples collected from XBP1s-TG and control mice 7 days after induction of *Xbp1s* expression. Based on our selection criteria (fold change ≥1.5 and a false discovery rate-corrected *p*-value, *p*_adj_ ≤ 0.05), 591 genes were differentially regulated (413 upregulated and 178 downregulated) in XBP1s-TG vs. control mice (Supplementary Fig. 5 and Supplementary Data 1). The heat map shows the top differentially regulated genes (Supplementary Fig. 5c). Notably, *Gpc6* expression was similar between groups, indicating that the transgene insertion has no obvious impact on *Gpc6* gene. Selected genes were validated by qRT-PCR analyses (Fig. 5a). Interestingly, RNA-Seq data indicated that the other 2 UPR sensors (ie, ATF6 and PERK) were significantly upregulated in XBP1s-TG mice. Indeed, Western blotting showed that protein levels of XBP1s and PERK were already increased on day 4 after Dox removal (Fig. 5b), and on day 8, protein levels of ATF6, PERK, and GRP78 remained markedly higher in the XBP1s-TG than in control mouse brains (Fig. 5c; Supplementary Fig. 6a). Moreover, the luciferase reporter assay suggested that XBP1s directly regulates the ATF6 and PERK promoters (Fig. 5d). Collectively, our data provided the in vivo evidence that the IRE1/XBP1s UPR branch is a master UPR regulator, which is in line with a previous cell culture study^16^, and also may reflect the fact that the IRE1 branch is the only major UPR branch conserved across species.

As expected, the pathway analysis of our RNA-Seq dataset revealed that the most enriched pathways are related to the UPR and ER stress (Fig. 5e). However, the XBP1s-regulated transcriptional landscape in neurons extended beyond canonical UPR targets, as some enriched pathways are linked to neuronal activity. For example, XBP1s may regulate the expression of ion channels and transporters, as well as genes that contribute to neurotransmitter-driven neuronal activity (Supplemental Data 1). In particular, the subunits (α4 and δ) of the post-synaptic GABA_A_ receptor, which have been proposed as potential new targets to treat seizures^17^, are downregulated in XBP1s-TG mouse brains. Interestingly, our analysis implied that extracellular signal-regulated kinase (ERK) signaling bridges between XBP1s and the GABA_A_ receptor (Fig. 5f). Indeed, phosphorylation of ERK1/2 was increased in XBP1s-TG mouse brains (Fig. 5b). It is important to note that among the differentially regulated genes (70% up- and 30% down-regulated), most of those associated with synaptic functions (e.g., *Gabra4*, *Gabrd*, and *CamkkII*) were downregulated (Supplementary Data 1). Changes in gene expression for all GABA_A_ receptor subunits, as revealed by our RNA-Seq analysis, are presented in Fig. 5g. Consistently, Western blot data also indicated a decrease in the protein level of GABRD, a key subunit of extra-synaptic GABA_A_ receptors, in XBP1s-TG hippocampal samples (Supplementary Fig. 6b). Taken together, we, therefore, speculated that downregulation of GABA_A_ receptor subunits in excitatory neurons leads to a loss of neural inhibition, thereby contributing to the seizure phenotype of XBP1s-TG mice.

### Impairment in spontaneous and tonic GABAergic inhibitory functions was found in *Xbp1s*-expressing neurons

**Fig. 6:**
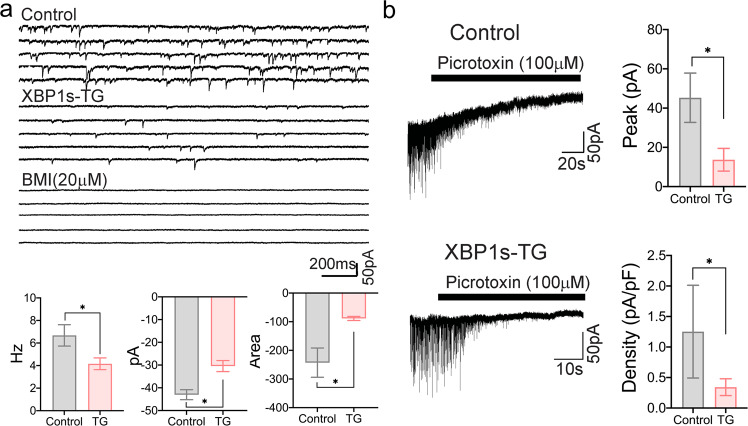
GABA_A_ receptor-mediated IPSCs and tonic currents. **a** Attenuated GABAergic inhibitory synaptic activity in neurons expressing *Xbp1s*. Representative bicuculline-sensitive GABA_A_-mediated sIPSCs traces recorded from control and XBP1s-TG neurons are shown in the top panels. Bar graphs for mean IPSCs frequency, mean IPSCs amplitude, and mean area (charger) under the sIPSCs, are shown in the bottom panels (Control: *n* = 10 neurons from 3 mice; XBP1s-TG: *n* = 13 neurons from 3 mice). **b** Reduction in extrasynaptic GABA_A_ receptor-mediated tonic currents in XBP1s-TG mice. Representative tonic current traces are shown in the 2 left panels. Bar graphs for tonic current amplitude and density between control (*n* = 13 neurons from 5 mice) and XBP1s-TG mice (*n* = 14 neurons from 5 mice) are shown in the 2 right panels. Data are presented as mean ± SEM. *p < 0.05. sIPSCs spontaneous inhibitory post-synaptic currents. BMI bicuculline methiodide.

Finally, we performed whole-cell patch clamp electrophysiology analysis to further dissect the cellular mechanisms. The observed changes in GABA_A_ post-synaptic receptor expression in our RNA-Seq analysis (Fig. 5) guided us to examine bicuculline-sensitive GABA_A_ receptor-mediated inhibitory synaptic transmission onto CA1 pyramidal neurons held at −70 mV (Fig. 6a). Compared to control neurons, XBP1s-TG neurons exhibited a significantly slower mean frequency of spontaneous inhibitory post-synaptic currents (sIPSCs) (*p* = 0.022), as well as smaller peak amplitude (*p* = 0.001) and areas (*p* = 0.003) (Fig. 6a). Thus, there was an overall decrease in inhibitory synaptic transmission onto *Xbp1s*-expressing neurons, through both direct modulation of synaptic inputs as well as indirect circuit effects at potentially pre-synaptic inhibitory interneuron level.

Alterations in spontaneous inhibitory synaptic activities led us to determine whether tonic inhibition mediated through extra-synaptic GABA_A_ receptors is also reduced in XBP1s-TG neurons (Fig. 6b). We found that the mean amplitude of the tonic current after picrotoxin was significantly smaller in XBP1s-TG vs. control neurons. The current density analysis for each neuron further revealed that the current density was also significantly smaller in XBP1s-TG vs. control neurons (*p* = 0.015). All data together indicated that *Xbp1s* overexpression impaired GABA_A_ receptor-mediated inhibitory function, which is expected to alter the balance of inhibition and excitation in post-synaptic transmission, in favor of the development of seizures.

## Discussion

Our data reveal a previously unrecognized link between XBP1s signaling and terminal seizure occurrence. Both RNA-Seq and electrophysiology analyses strongly indicate that GABA_A_ post-synaptic inhibitory function is substantially impaired if *Xbp1s* is sustainedly expressed in excitatory neurons, which likely constitutes a crucial mechanism responsible for the induction of seizure occurrences in XBP1s-TG mice. This conclusion is supported also by the beneficial effects of VPA treatment, which enhances GABA function^18^.

The striking phenotype observed in our XBP1s-TG mice is a highly unexpected finding, since prior *Xbp1s* overexpression studies showed an overall positive effect. For example, we reported that XBP1s-TG mice had improved stroke outcome^19^. In this previous work, we introduced stroke on day 7 after Dox removal and then analyzed the outcome the next day. These data thus indicate that short-term overexpression of *Xbp1s* in neurons is neuroprotective in stroke. Notably, 2 other studies in which *Xbp1s* was constitutively overexpressed in neurons also demonstrated primarily beneficial effects on cognitive function. One investigation showed that *Xbp1s* overexpression prevents the loss of dendritic spines and improves neuronal plasticity in a mouse model of AD^5^. In this study, *Xbp1s*-expressing viral vectors were delivered into the hippocampus and likely expressed in multiple cell types, as a universal promoter was used. In the other study, the authors generated a transgenic mouse line expressing *Xbp1s* under control of a prion promoter^11^. Although high levels of *Xbp1s* transgene expression were observed in neurons, this prion promoter is active in many non-neural cell types, including astrocytes^20^. In contrast, in our XBP1s-TG mice, a Camk2a promoter was used and thus, *Xbp1s* was expressed predominantly in forebrain excitatory neurons^21,22^.

Critically, some prior clinical observations implicate that dysregulation of *XBP1* can lead to severe alterations in brain function. For example, a microarray analysis of DNA samples from Japanese populations revealed that a mutation in the *XBP1* promoter impairs feedback regulation of *XBP1* expression, and may significantly increase the risk for bipolar disorder^12^. Of note, the finding from this report failed to be replicated in European populations^23^. Also, increased protein levels of XBP1s were found in dorsolateral prefrontal cortex samples collected at autopsy from schizophrenic patients^24^. Moreover, an analysis of resected tissue samples revealed that *XBP1* is overexpressed and activated in the hippocampus of patients with mesial temporal lobe epilepsy^25^. Interestingly, a clinical report described 3 individuals who developed various types of seizures in infancy, and showed failure to thrive^15^. Whole-exome analysis identified heterozygous *RNF13* gain-of-function mutations in these 3 individuals. Since RNF13 enhances IRE1 function, these mutations may also lead to increased expression of *XBP1s*. The authors attribute the clinical phenotype in the affected individuals to abnormally increased IRE1 UPR-induced apoptosis. However, in light of our findings here, increased *XBP1s* expression-mediated abnormal neuronal activity may play a role in the phenotype in these 3 individuals.

Our electrophysiology analysis provided important insight into cellular mechanisms underlying the observed seizure phenotype. The GABA_A_ receptor changes in XBP1s-TG mice may facilitate the transition to the recurrent seizure state. By specifically examining post-synaptic inhibitory functions in the *Xbp1s*-expressing neurons, our whole-cell physiology data revealed a strong reduction in GABA_A_ receptor-mediated inhibitory synaptic transmission. Such defects in GABAergic synaptic transmission may represent the neuronal mechanism underlying the seizure activity. Studies in epileptic animal models have reported that either spontaneous^26^ or tonic^17,27^ inhibition are reduced. In this regard, the diminished spontaneous GABA outflow and extrasynaptic neurotransmitter spillover or tonic inhibition following *Xbp1s* expression may be closely associated with an increased susceptibility to convulsions and eventually, to the development of seizures. Moreover, the reduction in tonic currents in *Xbp1s*-expressing neurons correlates well with the decreases in gene expression linked to the GABA_A_ receptor subunits (e.g., α4 and δ), as revealed by our RNA-Seq data.

Another interesting finding is that even at a late stage in seizure progression in XBP1s-TG mice, adding Dox back into the drinking water to block transgene *Xbp1s* expression can still completely reverse the severe phenotype, and prevent death. Thus, XBP1s-TG mice may be a unique and valuable genetic model of symptomatic seizures that can be precisely controlled in an inducible and reversible manner, a notable advantage over many current genetic animal models of spontaneous seizure development^28^. Hence, our mouse model reported here may be useful for investigating mechanisms responsible for seizure generation and for testing compounds for seizure management.

There are a few limitations to be discussed here. First, there is little information as to whether XBP1s regulates neuronal excitability. Here we show that *Xbp1s* overexpression in brain neurons modulates their electrophysiologic properties. Although our data suggest that these changes (e.g., tonic inhibition) are, at least partly, related to expression level of GABA_A_ subunits, other mechanisms may be also involved. For example, the RNA-seq data indicate that XBP1s is a central regulator of the UPR, a signaling pathway that engages protein folding and trafficking. Thus, *Xbp1s* overexpression may impact receptor trafficking. Indeed, one study showed that *Xbp1s* overexpression in HEK293T cells enhances ER folding capacity, which substantially promotes folding, trafficking, and functional surface expression of a misfolding-prone GABA_A_ α1 mutant^29^. Thus, XBP1s-mediated changes in the trafficking and surface distribution of neurotransmitter receptors are worth of further investigation. Second, VPA has a broad spectrum of mechanism of action, including enhancing GABAergic neurotransmission. Compounds that specifically target GABA_A_ receptors may be exploited in future studies to provide further mechanistic insight. Lastly, the clinical relevance of our findings should be cautiously implicated. Although our data indicate a link between the elevated expression level of *Xbp1s* to seizure occurrence, this link was derived from transgenic mice overexpressing *Xbp1s* in selected neurons. The effects of *Xbp1s* overexpression in other cell types on brain function need to be addressed.

In summary, a large body of evidence has indicated that activation of the XBP1s pathway is a protective response under various stress conditions, and IRE/XBP1s-based therapeutic treatments for brain disorders have been increasingly explored^30,31^. Cautiously, our results here demonstrated that sustained *Xbp1s* expression in a subset of excitatory neurons can cause dysfunctional neuronal activity, and lead to persistent seizures and inevitable death.

## Methods

### Animals

All experiments were conducted with protocols approved by the Duke University and Durham VA Medical Center Institutional Animal Care and Use Committees. These studies were conducted in accordance with the United States Public Health Service’s Policy on Humane Care and Use of Laboratory Animals. Both male and female mice were used. C57BL/6 mice were obtained from The Jackson Laboratory (Bar Harbor, ME). *Xbp1*^f/f^ mice and inducible XBP1s transgenic mice (TRE-XBP1s) were kindly provided by Dr. Laurie Glimcher^32^ and Dr. Joseph Hill^33^, respectively. We crossed TRE-XBP1s mice with Camk2a-tTA (JAX #007004) mice to generate double transgenic mice with inducible expression of *Xbp1s* in neurons: TRE-XBP1s;Camk2a-tTA mice (XBP1s-TG). In XBP1s-TG mice, *Xbp1s* expression is suppressed by doxycycline (Dox) presented in the drinking water. Upon Dox withdrawal, *Xbp1s* expression is activated. To generate neuron-specific *Xbp1* knockout mice, *Xbp1*^f/f^ mice were cross-bred with Emx1^Cre/Cre^ mice (JAX #005628) to obtain *Xbp1*^f/f^;Emx1-Cre (Xbp1-cKO) mice. To visualize the pattern of Cre expression controlled by the Camk2a promoter, CAG-SUMO mice (generated previously in our lab)^34^ were mated with Camk2a-Cre (JAX #005359). Note, all mouse lines were on a C57BL/6 genetic background. Primers for genotyping are listed in Supplementary Table 2.

### Transgene integration site analysis

Targeted locus amplification (TLA) was used to identify the integrated sites of the transgene vector in the TRE-XBP1s mouse line^35^. The TLA analysis was performed by Cergentis (Utrecht, Netherlands) using mouse splenocytes. Two primer sets targeting *Xbp1* were used: ATCCGGGCTTTCTTTCTATC and TGGTGGATTTGGAAGAAGAG, and CTGGTAAGGAACTAGGTCCT and AGCTGGAA GCCATTAATGAA. The mouse genome assembly GRCm39 was used as a reference.

### Behavioral tests

All data were acquired by a researcher blinded to the genotype and group assignments whenever possible. For behavioral studies, 2-4-month-old mice (both male and female) were used. Before each test, mice were habituated to the test room for at least 30 minutes.

#### Seizure scoring

The scoring chart that was used in the treatment experiment is shown in Supplementary Table 3. Seizure severity was scored on a scale of 6. Mice were constantly monitored for 8 hours a day.

#### Grip strength

This test was used to evaluate muscle strength of the forelimbs. The mouse was lowered over a grid and permitted to grip the top portion of the grid with its forepaws. The mouse was gently pulled from the grid by its tail. Grip strength was represented as the g-force required for the mouse to release its grip on the grid. This procedure was repeated 3 times and the mean grip strength was calculated for that session.

#### Social dominance tube test

This test was performed essentially as described^36^. Two mice were positioned at opposite ends of the tube and released simultaneously, and the latency time was recorded. The mouse that forced the opponent from the tube was designated the ‘winner’ or dominant mouse, whereas the other animal was designated as the ‘loser’ or subordinate. Positive or negative numbers were used to signify “won” or “lost” events, respectively.

### Continuous video-electroencephalography (cvEEG) recording

XBP1s-TG mice (2–3 months old; both male and female) were studied to assess electrographic and clinical seizures. Briefly, to implant epidural electrodes, mice were anesthetized with isoflurane, and then placed in a stereotaxic frame where 1-mm holes were drilled in the parietal bones for epidural EEG electrode placement. Three leads were placed: left side, right side, and ground lead, and the EEG leads were secured in place using instant hardening epoxy. The animals were allowed to recover for one week. On the day when Dox was withdrawn, animals were placed into the cvEEG monitoring system (Neurofax EEG-1200; Nihon Kohden, Shinjuku City, Tokyo, Japan). Animals were single-housed with *ad libitum* access to food and water. The epidural EEG leads were attached to the head box and cvEEG monitoring was performed for up to 21 days to characterize seizure development and frequency over time. cvEEG was recorded and analyzed on the Neurofax EEG-1200 and reviewed using Neuroworkbench V04-32 software (Nihon Kohden). Seizures were counted when the electrographic seizure activity had a video correlate of seizure behavior. Seizure behavior was scored on a modified Racine scale (mRacine)^37^. EEG artifacts that occurred when the animal was moving or scratching the leads were confirmed by video and were not counted. The reviewer of the cvEEG data was blinded to the genotype of the animals.

### RNA preparation and quantitative reverse transcription PCR (qRT-PCR)

Total RNA was extracted from frozen hippocampal tissues using TRIzol reagent (Invitrogen, Carlsbad, CA). To obtain highly-purified RNA samples for RNA-Seq analysis, total RNA was treated with DNase I and then purified using the RNeasy MinElute Cleanup kit (Qiagen, Hilden, Germany). A standard qRT-PCR procedure was followed. cDNA samples were prepared using the SuperScript III First-Strand Synthesis System (ThermoFisher Scientific, Waltham, MA). qRT-PCR was performed in a Lightcycler 2.0 (Roche Life Sciences, Indianapolis, IN). All primers are listed in Supplementary Table 2.

### RNA-Seq and data analysis

RNA preparation was performed as described above. Libraries representing 4 independent XBP1s-TG samples and 4 independent control samples were prepared and sequenced in the Genomic Analysis and Bioinformatics Shared Resource at Duke University Medical Center. The procedure was similar to the one used in our previous study^38^. Briefly, after assessment of the sample quality control, approximately 500 ng total RNA per sample was used for library construction with the KAPA Stranded mRNA-Seq Kit (Kapa BioSystems, Wilmington, MA). Libraries were indexed using a single indexing approach and sequenced in an Illumina HiSeq 4000 sequencing platform. After sequencing, Raw.bcl files were de-multiplexed and converted to fastq files using bcl2fastq2 v2.20. The raw data have been deposited in the GEO database with the accession number GSE160263.

To inspect and assure the quality of the sequence data generated, the FastQC tool was used followed by the use of the Trimmomatic tool to remove adaptor sequences that might be present (TruSeq3-PE.fa:2:30:10) and to remove both 5’ nucleotide bias (HEADCROP:12) and low quality sequence (TRAILING:20, SLIDINGWINDOW:4:20, MINLEN:15). Surviving read pairs per library were then mapped against the current instance of the mouse genome (GRCm38) using the “RNA-Seq” tool supported within the CLCbio Genomics Workbench under default parameters. Thereafter, expression per known gene (GRCm38.83.gtf) was enumerated in TPM (Transcript Per Million) units. These data were imported into R, and pedestalled by 2, Log2 transformed, filtered to remove genes not having at least one transformed value >1, and quantile normalized. Post normalization, the quality of the data was confirmed via Tukey box plot, covariance-based PCA scatter plot, and Pearson correlation heat map. To remove noise-biased expression, the lowest modeling was performed by library type (CV ~ mean expression) and the resulting fits were inspected for the lowest consensus expression value where the linear relationship between CV and mean expression was grossly lost. Expression values for a gene less than this value were construed to be noise-biased and floored to equal this value. Genes that did not have at least one library with a post-floored expression greater than this value were discarded as non-informative. For surviving genes, the Welch-modified t-test was applied under Benjamini-Hochberg false discovery rate multiple comparison correction conditions to identify those having dysregulated expression between XBP1s-TG and control. Test results were summarized by volcano plot using color and symbols with dysregulated genes identified as those having both a corrected *p* < 0.05 and an absolute linear fold difference of means ≥1.5X. To describe the expression across libraries for the top 100 dysregulated genes identified, a scaled heat map was generated using the heatmap.2() function. Corresponding enriched functions and pathways for all dysregulated genes were identified using the Ingenuity Pathway Analysis (IPA) program.

### Western blotting, immunohistochemistry, and Nissl staining

Western blotting was performed based on a standard protocol. Immunofluorescence staining was performed on frozen tissues, as described previously^39^. In short, mouse brains were fixed by transcardial perfusion with 4% paraformaldehyde, and stored at −80 °C. Frozen brain sections (25 μm thick) were obtained using a Leica cryostat, and immunostained using a free-floating staining method. All primary antibodies used in this study are listed in Supplemental Table 4. For Nissl staining, paraffin-embedded sagittal brain sections (5 μm) were stained with 0.1% cresyl violet solution. Images were captured on a Zeiss Axio Imager Z2 motorized fluorescence microscope (Carl Zeiss MicroImaging).

### Luciferase reporter assay

This assay was performed as described^40^. HEK293T cells were seeded into 24-well plates, and 24 hours later, were transfected with different combinations of plasmids. Three firefly luciferase reporter plasmids with different promoters were evaluated. The vectors 5XATF6-luc containing 5 designed ATF6-binding elements, and the ATF6 promoter-luc containing the DNA segment of −1500 to −1 base pairs relative to the transcriptional start site of the ATF6 gene, were kindly provided by Dr. Wek^41^. The vector PERK promoter-luc containing the 1802-bp DNA segment upstream of the transcription start site of the PERK gene, and the IRE1α promoter-luc containing the 1500-bp DNA segment upstream of the transcription start site of the IRE1α gene, were kindly provided by Dr. Nakayama^42^. The XBP1s expression vector (CMV-XBP1s) was generated in our lab using the pcDNA3.1 vector. The Renilla luciferase plasmid phRL-TK (Promega) was co-transfected as an internal control. A plasmid mixture of CMV-XBP1s or CMV-null (200 ng), a firefly luciferase reporter with different promoters (100 ng), and phRL-TK (10 ng) were transfected into cells. After 48 hours, luciferase activity was measured using the Dual-luciferase assay reporter system (Promega) and calculated as previously described^40^.

### Pharmacologic treatments

Mice were randomly assigned to each group. We used 2 classic anti-epilepsy drugs: carbamazepine (CBZ) and valproic acid (VPA). The treatments were initiated on day 10 after Dox withdrawal from the drinking water, the time-point at which the seizure activity began to become obvious. CBZ was dissolved in DMSO and diluted in 0.9% NaCl. VPA was directly added to the drinking water of both control and transgenic groups. The dosages were based on previous studies^43^. For CBZ, we increased the dose daily to mimic the clinical application (Day 10: 5 mg/kg; Day 11: 10 mg/kg; Day 12: 15 mg/kg; Day 13: 20 mg/kg; Day 14: 25 mg/kg). For VPA, to reach 500 mg/kg per day, we decided the VPA concentration in the drinking water on the average daily volume of water consumed (around 5 mL per day).

### Measurement of glucose in the brain

Brain tissue (one hemisphere) was homogenized with 1 mL Trizol and 0.2 mL chloroform, and centrifuged at 16,000 x *g*. The aqueous supernatant was collected, and stored at −70 ^o^C^19^. For glucose measurement, 20 μL of brain extract was mixed with 50 μL of derivatization solution (see below) with 5 μg/mL glucose-^13^C_6_ (internal standard), and then vortexed and incubated at 75 ^o^C for 30 minutes. After centrifugation, the supernatant was injected into an LC/MS/MS system. To prepare the derivatization solution, 12.5 mg PFBHA (Sigma) was dissolved in 1.5 mL methanol, then 2.5 mL acetonitrile and 1 mL of 200 mM ammonium acetate were added, and the pH was adjusted to 4.0 using glacial acetic acid.

The samples were analyzed on Shimadzu 20 A series LC system interfaced with Applied Biosystems/SCIEX API 4000 QTrap MS/MS and Analyst (version 1.6.1) software. A Phenomenex Kinetex (#AJ0-4287), 3 × 4 mm RP C18 guard column at 40 ^o^C was used for separation. Mobile phase A: 0.1% formic acid and 2% acetonitrile in MS-grade water; mobile phase B: acetonitrile; flow rate: 0.7 mL/min, 1:1 MS:waste split. Elution gradient: 0–0.5 min 1–50% B, 0.5-1 min 50% B, 1–1.2 min 50–1% B; run time: 3 min. The pure standards, glucose and glucose-^13^C_6_ (internal standard) were individually infused as 100 ng/mL solutions in 50%A/50%B at 10 µL/min flow rate and ionization and ion path parameters were optimized to provide maximal ion count for “parent” and collision-produced (“daughter”) MS/MS transitions. Parent/daughter quantifier [qualifier] ions utilized: 373.9/148.7[166.7] for glucose and 379.9/153.6[166.7] for glucose-^13^C_6_. Calibration curve: 0.4–150 μg/mL glucose in Trizol:water (1:2); mobile phase A (r^2^ = 0.998). The lower limit of quantification (LLOQ) at >80% accuracy: 0.4 μg /mL.

### Photoacoustic microscopy (PAM)

Oxygen saturation of hemoglobin (sO_2_) in the mouse brain was imaged by a customized photoacoustic (PA) microscopy system, which was composed of a high-frequency (50 MHz) ultrasound transducer (V358-SU; Olympus, Shinjuku, Japan) and 2 pulsed lasers with different wavelengths (532 & 640 nm). The 640-nm excitation light source was a Credo dye laser (DCM; Sirah, Grevenbroich, Germany) pumped by a pulsed laser at 532 nm (IS8II-E; EdgeWave, Würselen, Germany), and the 532-nm light source was a Nd: YAG fiber laser (VPFL-G-20; V-Gen, Tel Aviv, Israel). The 2 beams were collimated and made coaxial before being focused by an objective lens (AC127-050-A; Thorlabs, Newton, NJ), which were triggered with a 2-μs delay for separating the excited PA signals. The PA signals were focused to the ultrasound transducer by an acoustic lens (#48-266; Edmund Optics, Barrington, NJ) and sampled at 500 MHz by a high-speed DAQ card (ATS9350;, AlazarTech, Pointe-Claire, Quebec, Canada). A two-dimensional motorized stage was used for raster scanning imaging of the whole mouse head (fur was shaved before imaging). During imaging, the temperature of the mouse was held at 37 °C by a heating pad and the mouse was anesthetized using isoflurane (1.5%). The sO_2_ levels in the mouse skull vessels were quantified by a traditional linear spectral unmixing method based on the different optical absorption spectra of HbO_2_ and HbR and the detected PA signals. The average sO_2_ level in a mouse brain was calculated by adding the sO_2_ levels, and dividing this by the total vessel pixels, and the distribution ratio of vessels with low (<80%) and high (>80%) sO_2_ levels was calculated by the corresponding vessel pixels divided by the total vessel pixels.

### Electrophysiology and data analysis

Control and XBP1s-TG mice (2–3 months old; both male and female) were placed on regular drinking water for 10–11 days (ie, without Dox) to induce *Xbp1s* expression.

#### Brain slice preparation

Mice were deeply anesthetized with isoflurane, and rapidly decapitated. The brain was quickly removed from the skull and immediately chilled in an ice-cold, oxygenated artificial cerebrospinal fluid (aCSF) solution containing the following (in mM): NaCl 120, KCl 3.3, NaHCO_3_ 25, NaH_2_PO_4_H_2_O 1.23, CaCl_2_ 1.8, MgSO_4_ 1.2, and glucose 10. Coronal slices (300 μm thick) containing the hippocampi were cut with a moving-blade microtome (Vibratome, 100 Plus, St. Louis, MO), and the slices were kept in oxygenated aCSF at 35 °C for 60 minutes and then maintained at room temperature until used for recording.

#### Whole-cell patch clamp recordings

Single slices were transferred to a recording chamber that was constantly perfused (∼3 mL/min) with oxygenated aCSF at 35 °C. The CA1 neurons were visualized under a Zeiss upright microscope (40 X water-immersion objective) and an enhanced differential interference contrast (DIC) video microscope system. Recording pipettes with the resistance of 3–5 MΩ were pulled from borosilicate glass capillaries (1.5 mm outer diameter) using a P97 electrode puller (Sutter Instruments, Novato, CA). Access resistance and input capacitance were electronically compensated by ∼60–70% and monitored throughout the experiment to confirm the stability of the recording.

Fast spontaneous inhibitory postsynaptic currents (sIPSCs) mediated by the GABA_A_ receptor were recorded from hippocampal CA1 pyramidal neurons held at −70 mV in the presence of 2-amino-5-phosphonovaleric acid (APV)(50 μM), 6-cyano-7-nitroquinoxaline-2,3-dione (DNQX) (20 μM), and CGP55845A (1 μM). The internal solution contained the following (in mM): Cs-gluconate 130, CsCl 10, EGTA 0.2, Mg ATP 4, Tri-GTP 0.3, HEPES 10, and QX-314, 4 The pH was adjusted to 7.4 with CsOH, and the osmolarity was 290 mOsm. In all instances, the recordings of spontaneous GABAergic IPSCs usually began at least 5 min after a whole-cell configuration was established with a stable baseline. Spontaneous IPSCs were completely blocked with bath-applied bicuculline methiodide (BMI, 20 μM), confirming that they are mediated by GABA_A_ receptors. Tonic currents were isolated after bath application of the GABA_A_ receptor antagonist picrotoxin (100 μM).

Data were recorded with a MultiClamp 700B amplifier, filtered at 10 kHz, and digitized at 20 kHz through a Digidata 1440 interface controlled by pClamp10.7 software (Molecular Devices, CA). Both the frequencies and amplitudes of CA1 sIPSCs were analyzed using Clampfit 10.7 software, and the threshold for detecting sIPSCs was used and followed by visual inspection to ensure the accuracy of detection.

### Statistics and reproducibility

The sample size for each experiment was based on previous or pilot studies. Randomization and blinding were implemented in experiments whenever possible. All measurements were collected from biological replicates. Statistical analyses were performed using Clampfit 10.7, Origin pro (Microcal Software), and Prism 8 (GraphPad Software). To compare 2 groups, statistical analysis was assessed by unpaired Student’s *t*-test. To compare more than 2 groups, one-way or two-way ANOVA with *post hoc* Holm-Sidak corrections for multiple comparisons were performed. The body weight and grip strength were analyzed with repeated-measures ANOVA (RMANOVA) with *post hoc* Bonferroni-corrected pairwise comparisons. Mantel-Cox log-rank test was used to analyze survival rates. The results are presented as mean ± SEM or the median. Statistical significance was set at *p* < 0.05.

### Reporting summary

Further information on research design is available in the Nature Portfolio Reporting Summary linked to this article.

## Supplementary information

Supplementary Information

Description of Additional Supplementary Files

Supplementary Data 1

Supplementary Data 2

Supplementary Movie 1

Supplementary Movie 2

Reporting Summary

## Data Availability

All data are included in the article and supplementary information. The RNA-Seq raw data are available at the GEO database repository with the accession number GSE160263. Source data of figures are provided in Supplementary Data 2. The unedited blots are shown in Supplementary Fig. 7. The data that support the findings of this study are available from the corresponding author upon reasonable request.
