# Supplementary material for: Sustained overexpression of spliced X-box-binding protein-1 in neurons leads to spontaneous seizures and sudden death in mice
Source: Commun Biol. 2023 Mar 9;6:252. doi: 10.1038/s42003-023-04594-8 (PMC9998612; doi:10.1038/s42003-023-04594-8)
Supplement: Supplementary file 1 — Supplementary Information [file 42003_2023_4594_MOESM1_ESM.pdf]

Supplemental Material

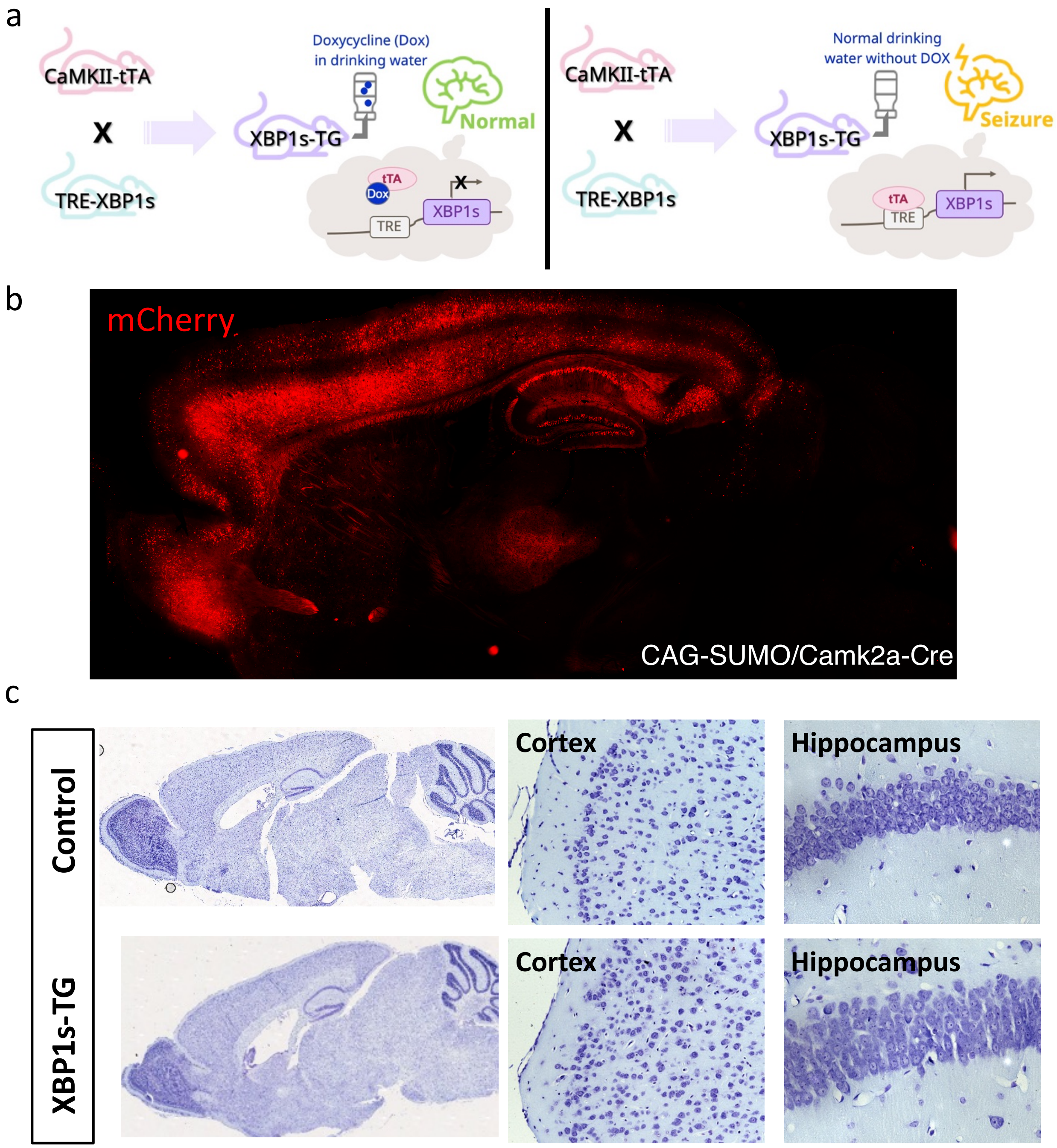

Supplemental Figure 1. XBP1s-TG mice. a) Schematic diagram of XBP1s-TG mice. In XBP1s-TG mice, neuron-specific expression of *Xbp1s* is controlled by a Camk2a promoter and a Tet-off system. Thus, *Xbp1s* expression was suppressed by doxycycline (Dox) and induced by changing to regular drinking water (without Dox). **b)** To examine the expression pattern driven by the Camk2a promoter, we crossed our CAG-SUMO

mouse line with Camk2a-Cre to generate CAG-SUMO/Camk2a-Cre mice. The mCherry signal depicts activation of the Camk2a promoter, indicating a widespread expression pattern in the forebrain. **c)** Nissl-stained sections of control and XBP1s-TG mouse brains. Mice were treated were kept on drinking water without Dox for 8 days.

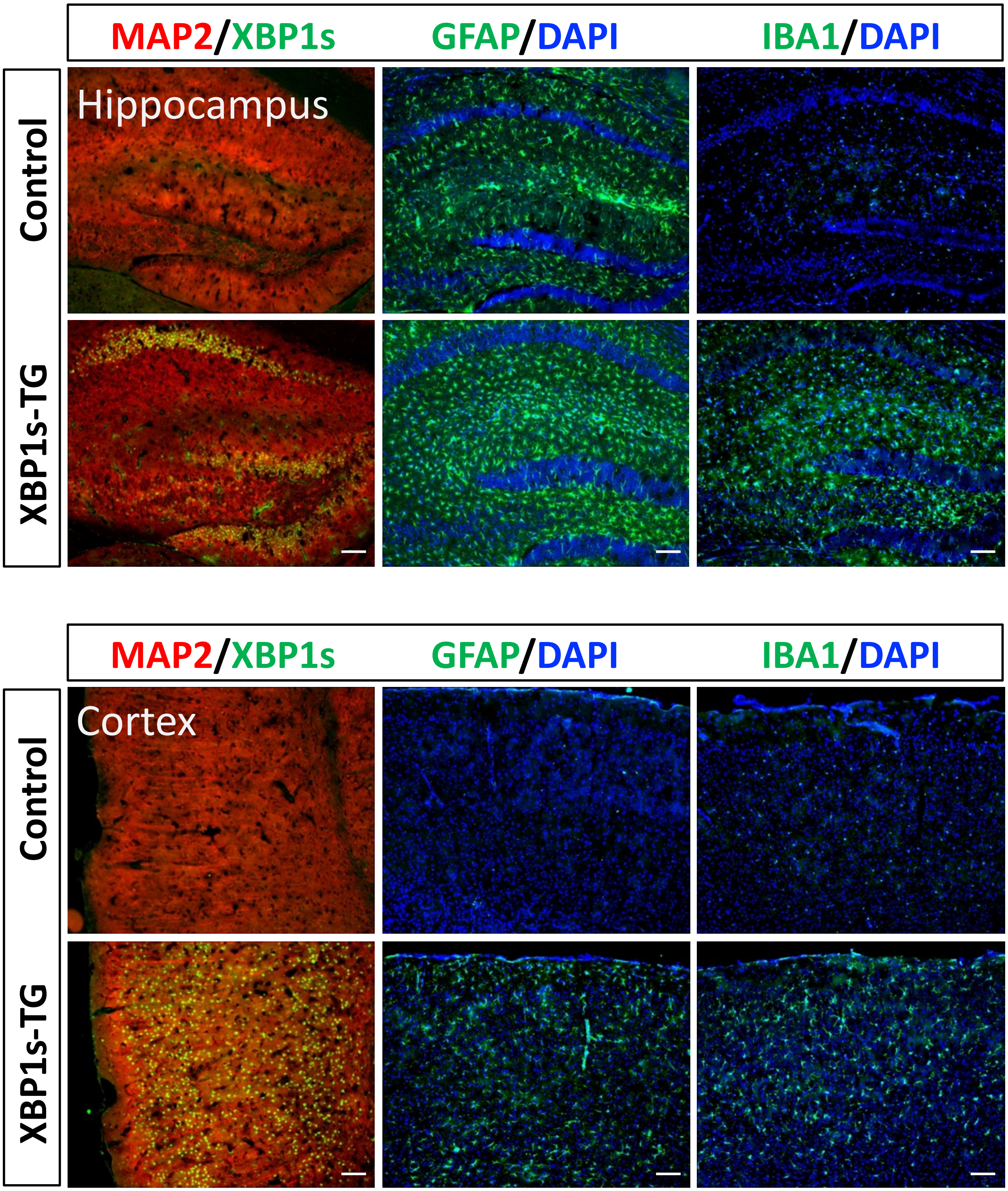

Supplemental Figure 2. Neuroinflammation was evident in the XBP1s-TG mouse brain at the late stage after induction. By day 13 of removal from DOX and induction of *Xbp1s* expression, astrocytes and microglia were massively activated in XBP1s-TG mouse brains. Representative images were shown here. Scale bar: 100 μm.

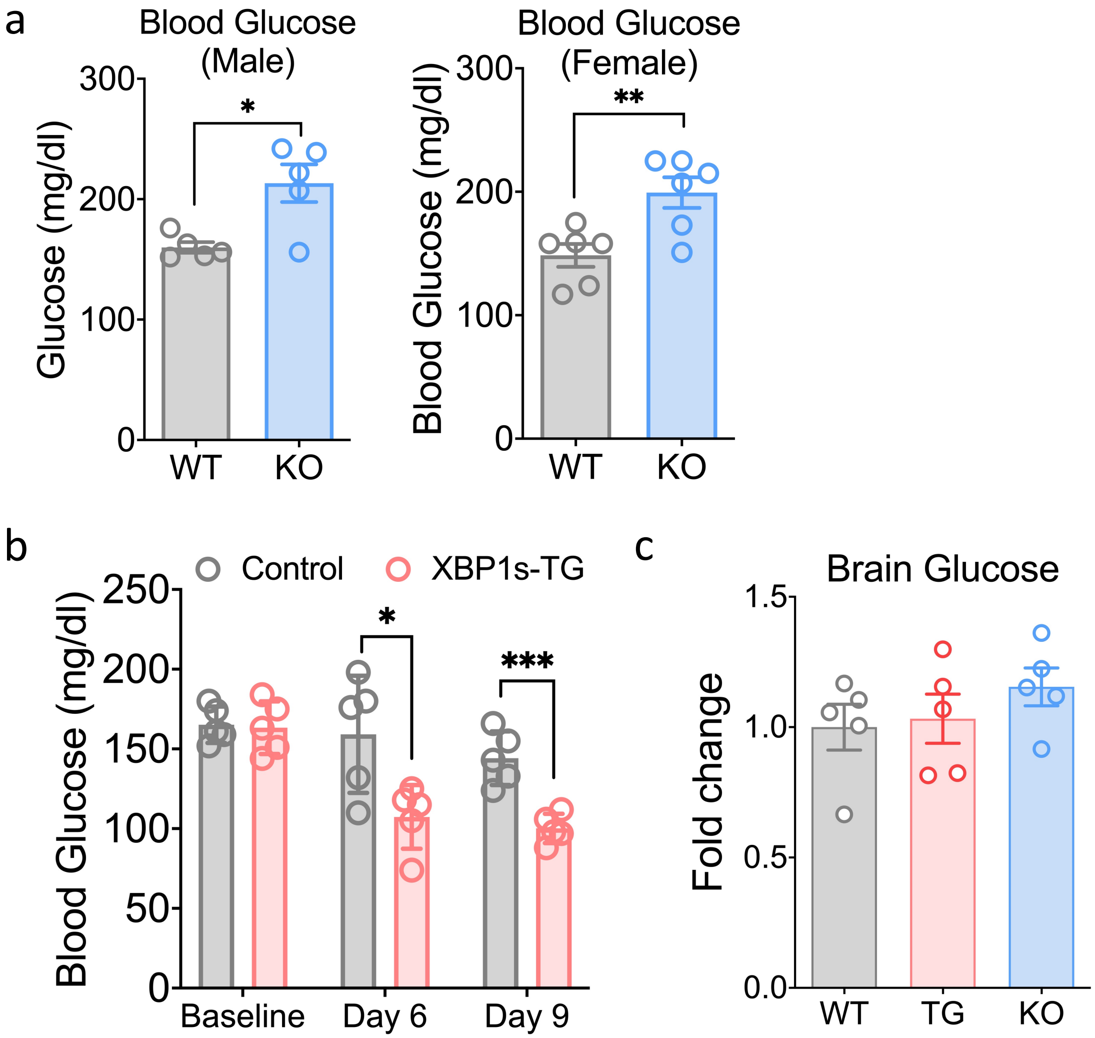

Supplemental Figure 3. Blood and brain glucose levels in Xbp1-cKO and XBP1s-TG mice. a) Blood glucose levels in Xbp1-cKO mice. Tail blood glucose levels in male (*left*) and female (*right*) wild-type (WT) and Xbp1-cKO (KO) mice (n = 5-6/group) were measured with a glucometer (CVS pharmacy) after 5 hours of food deprivation. **b)** Blood glucose levels in XBP1s-TG mice (n = 5/group). **c)** Brain glucose levels detected by LC-MS for WT, XBP1s-TG (TG), and Xbp1-cKO (KO) mice. Before the measurement, XBP1s-TG mice were kept on drinking water without Dox for 7 days.

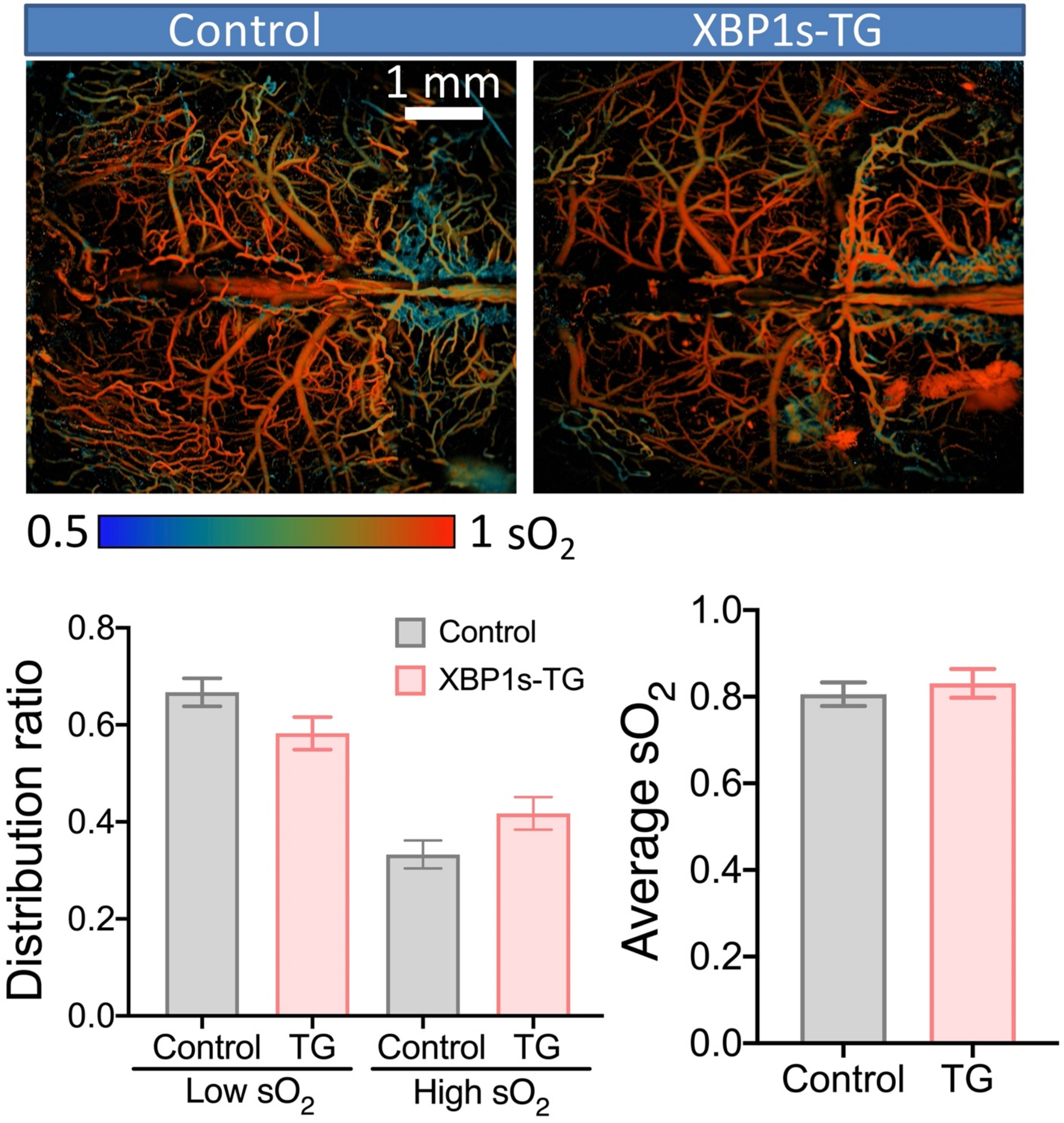

Supplemental Figure 4. Brain sO_2 levels. On day 12 after removal of Dox from the drinking water to induce expression of *Xbp1s*, control (n = 8) and XBP1s-TG (n = 3) mice were subjected to PAM imaging to examine changes in sO_2 in intact brains. Top: representative PAM sO_2 images; bottom: quantification data of sO_2 levels.

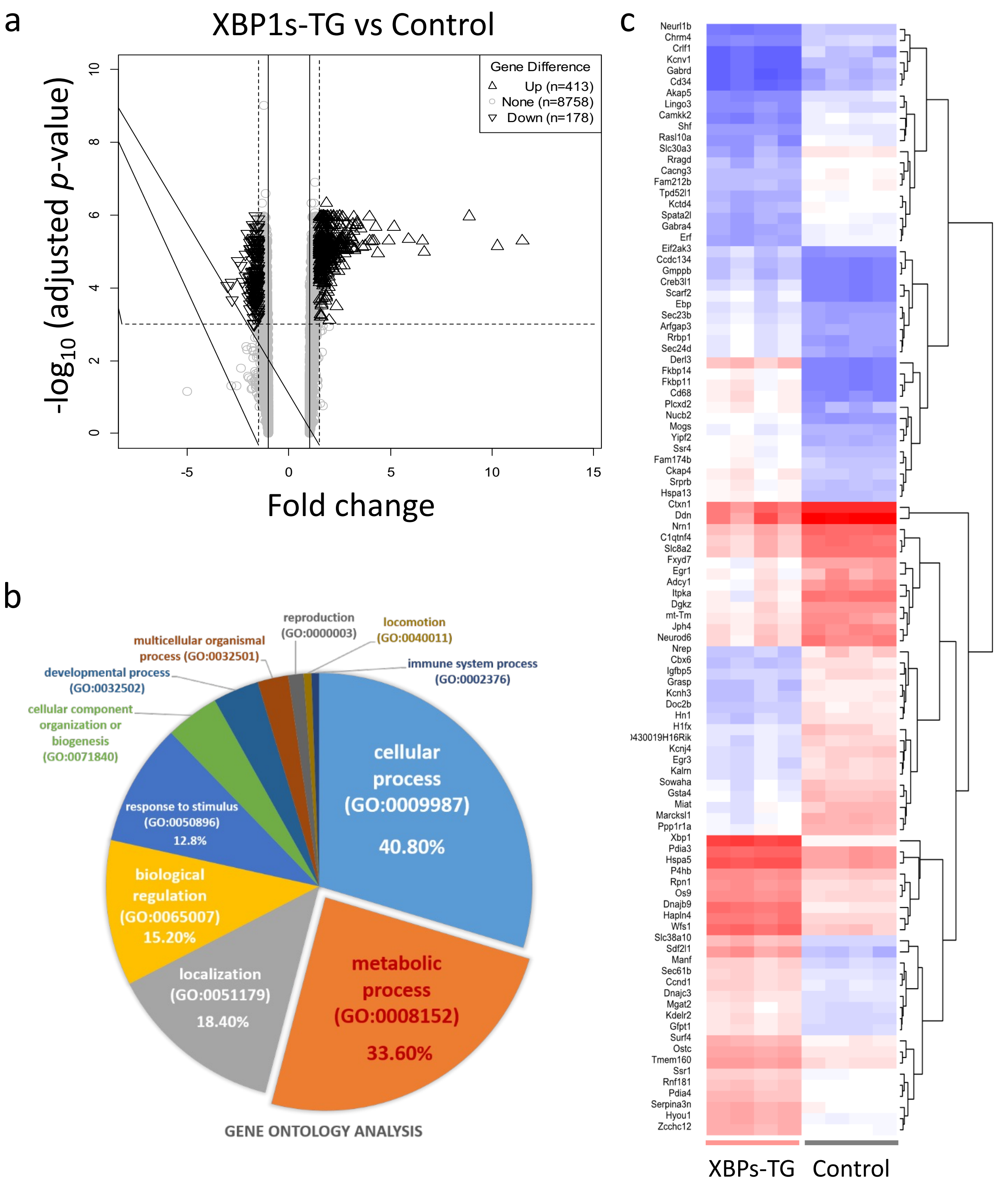

Supplemental Figure 5. RNA-seq analysis. a) Volcano plot. This plot depicts distribution of significance [-$\log_{10}$ (adjusted *p*-value)] vs fold-change [$\log_2$ (fold change)] for all genes. **b)** Gene ontology analysis of differentially regulated genes using Panthers online software. **c)** Heat-map of differentially expressed genes between control and

XBP1s-TG mice. Differentially regulated genes (XBP1s-TG vs control; Supplemental Data 1) were used to generate the heat map. Heat map colors indicate the extent of fold changes between XBP1s-TG vs control samples.

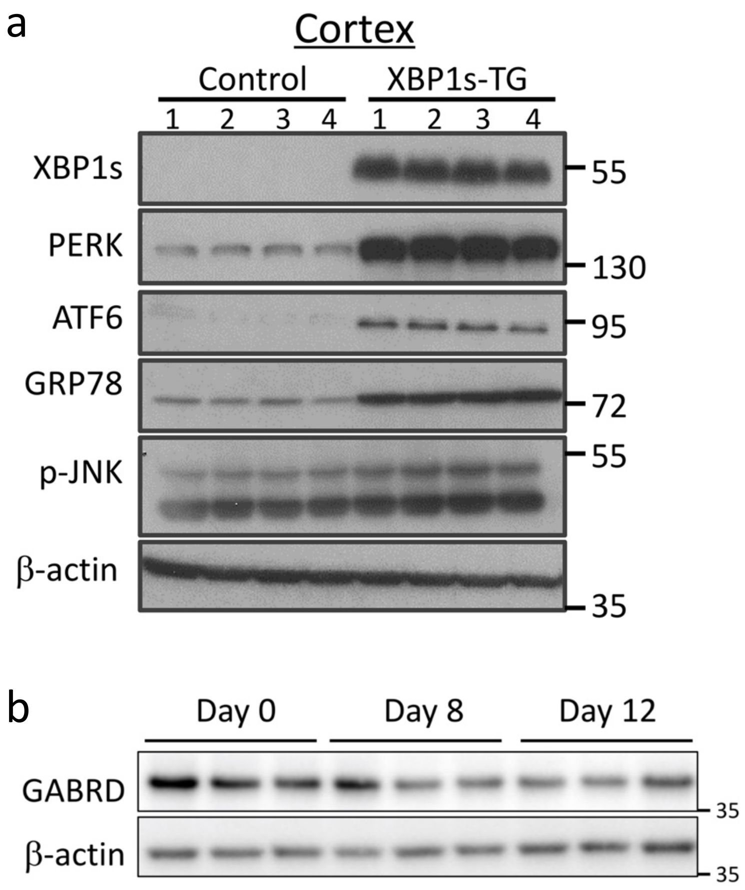

Supplemental Figure 6. Western blotting analysis of XBP1s-TG mouse brain samples. a) Key components of the UPR. Cortex samples were collected on day 8 after Dox removal from the drinking water. **b)** $GABA_A$ receptor δ subunit. Hippocampal samples of XBP1s-TG mice were collected on the indicated day after Dox removal from the drinking water.

Fig. 5b

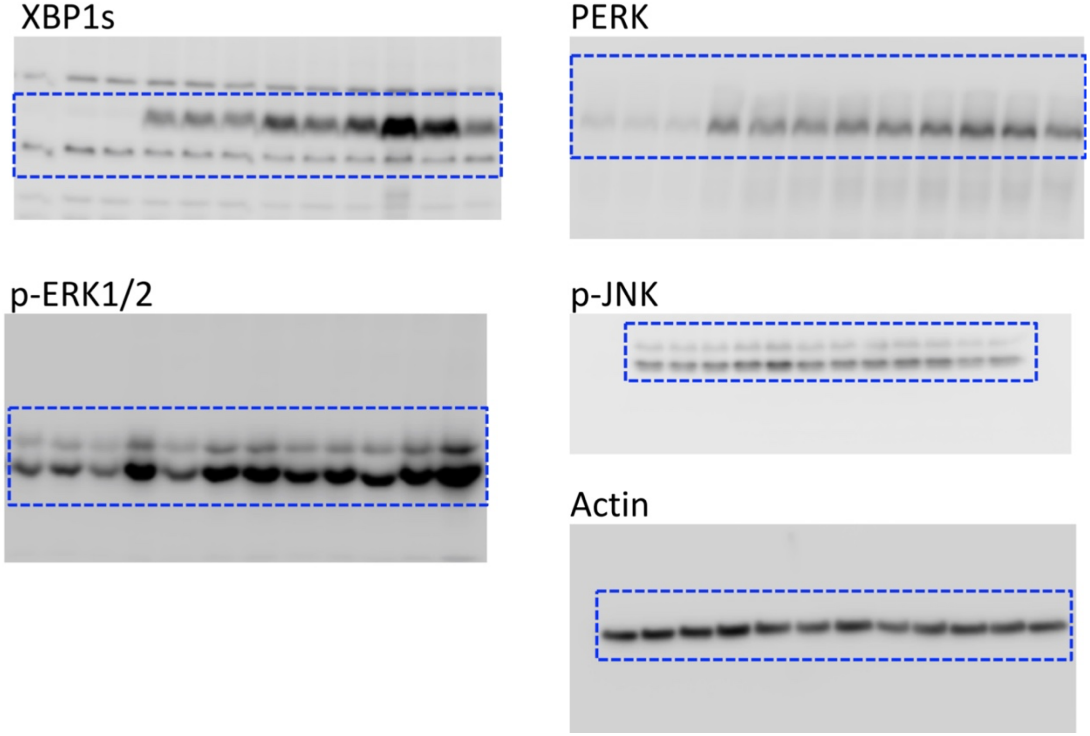
XBP1s
PERK
p-ERK1/2
p-JNK
Actin

Fig. 5c

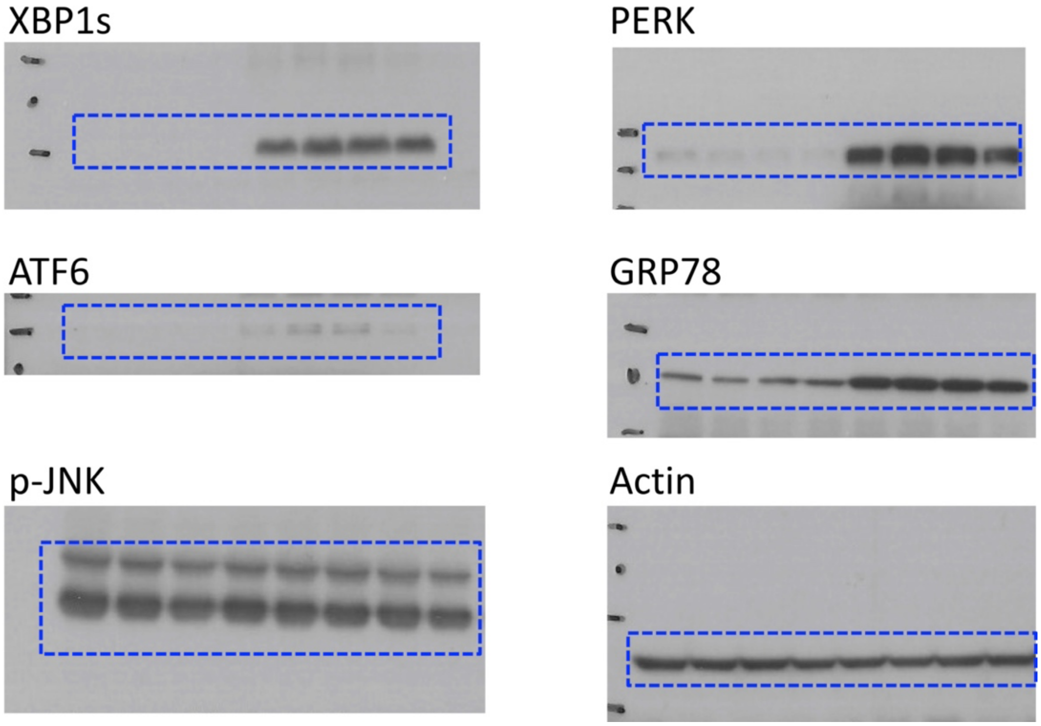
XBP1s
PERK
ATF6
GRP78
p-JNK
Actin

Supplementary Fig. 6a

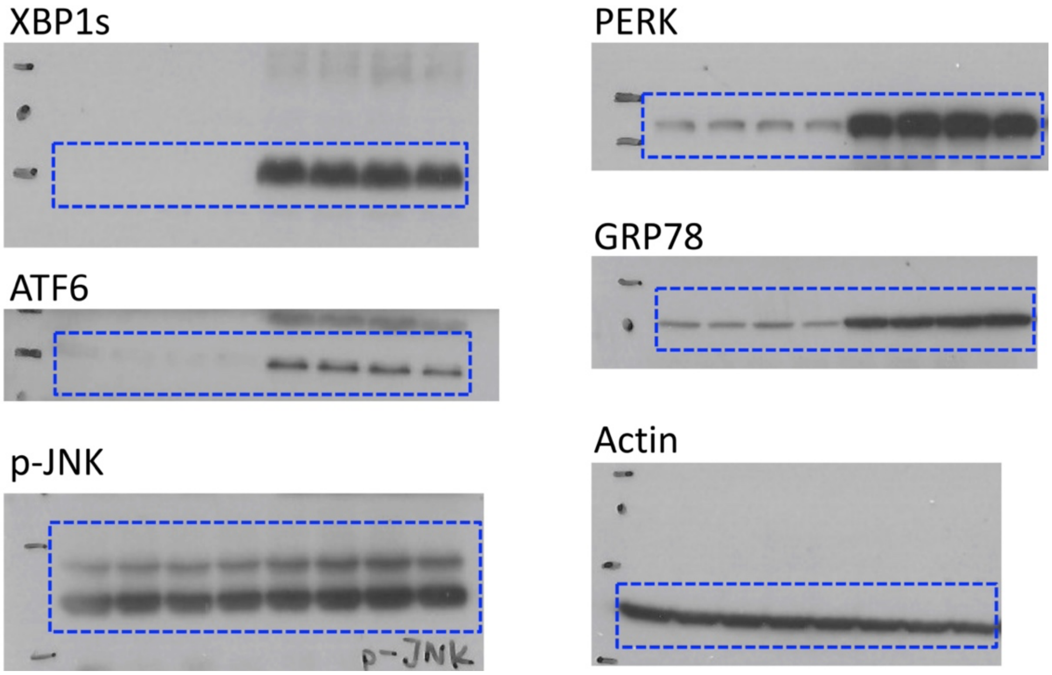

Supplementary Fig. 6b

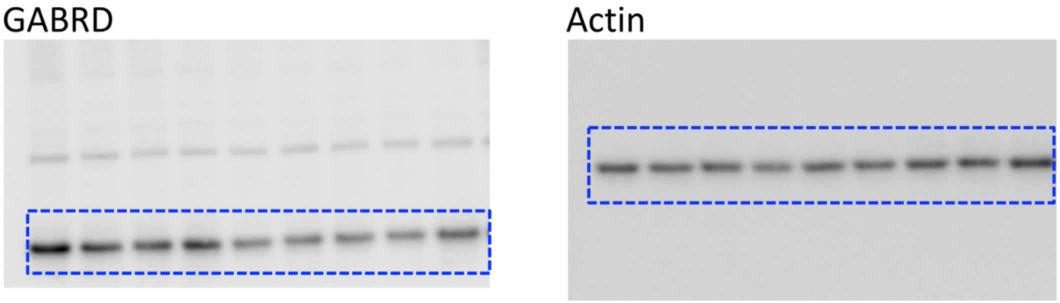

Supplemental Figure 7. The unedited Western blots.

Supplemental Table 1. Arterial blood gas in control and XBP1s-TG mice.

	Control	XBP1s-TG	*p* (n=4)
pH	7.414±0.018	7.358±0.028	0.136
pCO_2 (mmHg)	29.40±3.13	31.65±1.64	0.548
pO_2 (mmHg)	166.0±9.82	169.0±11.10	0.846
HCO_3 (mmol/L)	18.60±1.31	17.78±0.80	0.611
TCO_2 (mmol/L)	19.75±1.44	19.00±0.82	0.666
Na^+	144.8±0.75	143.5±0.65	0.254

Blood samples were collected from XBP1s-TG mice 7 days after Dox removal from the drinking water.

Supplemental Table 2. List of PCR primer sequences.

Purpose	Gene	Primer sequences (5'→3')
Genotyping	floxed-Xbp1	Forward: ACTTGCACCAACACTTGCCATTTC<br>Reverse: CAAGGTGGTTCACTGCCTGTAATG
	Cre	Forward: GGTCGATGCAACGAGTGATGAGG<br>Reverse: GCCAGATTACGTATATCCTGGCAG
	tTA	Forward: CGCTGTGGGGCATTTTACTTTAG<br>Reverse: CATGTCCAGATCGAAATCGTC
	Xbp1s	Forward: ACACGCTTGGGAATGGACAC<br>Reverse: CCATGGGAAGATGTTCTGGG
qRT-PCR	*Atf6*	Forward: GGTTCTTCCTCATGGACCAG<br>Reverse: CAGCTGTCGCCATATAAGGAA
	CaMKKII	Forward: GCTTTCTGGTCGCAAGATGT<br>Reverse: TTGGACAGCACTTTCATTGC
	Derl3	Forward: TCAACTTCTTCGGCTTACTCAAC<br>Reverse: GGGAAGGGGCAGGTAATCG
	Gaba4a	Forward: CCCTCCTGCACTTCCTGTG<br>Reverse: TTGTCAATCCATGTCTGTCTGA
	Gabad	Forward: GCTCCTGCTGCTCTGCAC<br>Reverse: AGGCCTAAGGTCTCGTTGGT
	GRP78	Forward: CGTATGTGGCCTTCACTCCT<br>Reverse: TTTCTTCTGGGGCAAATGTC
	GFAT1	Forward: TAAGGAGATCCAGCGGTGTC<br>Reverse: CAGCTGTCTCGCCTGATTGA
	Perk	Forward: GCGTCGGAGACAGTGTTTG<br>Reverse: CGTCCATCTAAAGTGCTGATGAT
	β-actin	Forward: TAGGCACCAGGGTGTGATG<br>Reverse: GGGGTGTTGAAGGTCTCAAA
Integration site analysis	F1	CTGGCGTAATAGCGAAGAGG
	R1	GGGGATCTTTTGTCTTTGGA

	F2	GGAGGGGTCAAGGAGATGAT
	R2	GATCCCTCGAGGAGCTTTTT

Supplemental Table 3. Seizure scoring chart.

Seizure Score	Behavioral Description
1	Immobile
2	Rigidity (Stretched forelimbs or erected tail)
3	Time of seizure onset noted (Movement of forelimbs above the ground rapidly)
4	Rearing (Forelimbs lift so high that mice fall over because of loss of balance)
5	Constant seizure (Persistent and continuous rearing that may lead the mice in a constant status and lay on the ground)
6	Jump (Suddenly jumps around intensely)

Supplemental Table 4. List of primary antibodies.

Antibody (Cat. #)	Dilution	Species	Manufacturer
ATF6 (sc-22799)	1:1000	Rabbit	Santa Cruz
p-ERK1/2 (4370)	1:1000	Rabbit	Cell Signaling Technology
GRP78 (3183)	1:1000	Rabbit	Cell Signaling Technology
p-JNK (4668)	1:1000	Rabbit	Cell Signaling Technology
PERK (3192)	1:1000	Rabbit	Cell Signaling Technology
XBP1 (SC-7160)	1:1000	Rabbit	Santa Cruz
GABRD (868A-GDN)	1:1000	Rabbit	PhosphoSolutions
β-actin (A3854)	1:10000	Mouse	Sigma
