# Supplementary material for: Sustained overexpression of spliced X-box-binding protein-1 in neurons leads to spontaneous seizures and sudden death in mice
Source: Commun Biol. 2023 Mar 9;6:252. doi: 10.1038/s42003-023-04594-8 (PMC9998612; doi:10.1038/s42003-023-04594-8)
Supplement: Supplementary file 2 — Description of Additional Supplementary Files [file 42003_2023_4594_MOESM2_ESM.pdf]

Description of Additional Supplementary Files

File name: Supplementary Data 1

Description: The list of differentially expressed genes (DEGs) of the RNA-Seq analysis of control and XBP1s-TG mouse hippocampi. The selection criteria are: $p_{adj} < 0.05$ and fold change ≥ 1.5 or ≤ -1.5.

File name: Supplementary Data 2

Description: The numerical source data for the graphs in the paper.

File name: Supplementary Movie 1

Description: This representative video shows behavioral expression of spontaneous seizures at the early stage after induced Xbp1s expression in a XBP1s-TG mouse.

File name: Supplementary Movie 2

Description: This representative video shows behavioral expression of spontaneous seizures at the later stage after induced Xbp1s expression in a XBP1s-TG mouse.
